# Supplementary material for: Neural Stem Cells Transplanted into Rhesus Monkey Cortical Traumatic Brain Injury Can Survive and Differentiate into Neurons
Source: Int J Mol Sci. 2024 Jan 29;25(3):1642. doi: 10.3390/ijms25031642 (PMC10855641; doi:10.3390/ijms25031642)
Supplement: Supplementary file 1 [file ijms-25-01642-s001.zip › ijms-2797803-supplementary.pdf]

Neural Stem Cells Transplanted into Rhesus Monkey Cortical Traumatic Brain Injury Can Survive and Differentiate into Neurons

Shuyi Liu [1,2,†], Liping Shi [1,2,†], Tianzhuang Huang [1,2], Yuyi Luo [1,2], Yongchang Chen [1,2,*], Shangang Li [1,2] and Zhengbo Wang [1,2,*]

[1] State Key Laboratory of Primate Biomedical Research, Institute of Primate Translational Medicine, Kunming University of Science and Technology, Kunming 650500, China; lpbrliushuyi@163.com (S.L.); sleeping0527@163.com (L.S.); huangtz@lpbr.cn (T.H.); luoyuyi2021@163.com (Y.L.); lis101@163.com (S.L.)

[2] Yunnan Key Laboratory of Primate Biomedical Research, Kunming 650500, China

* Correspondence: chenyc@lpbr.cn (Y.C.); wangzb@lpbr.cn (Z.W.); Tel.: +86-13508715773 (Z.W.)

† These authors contributed equally to this work.

Supplemental Figure and legend

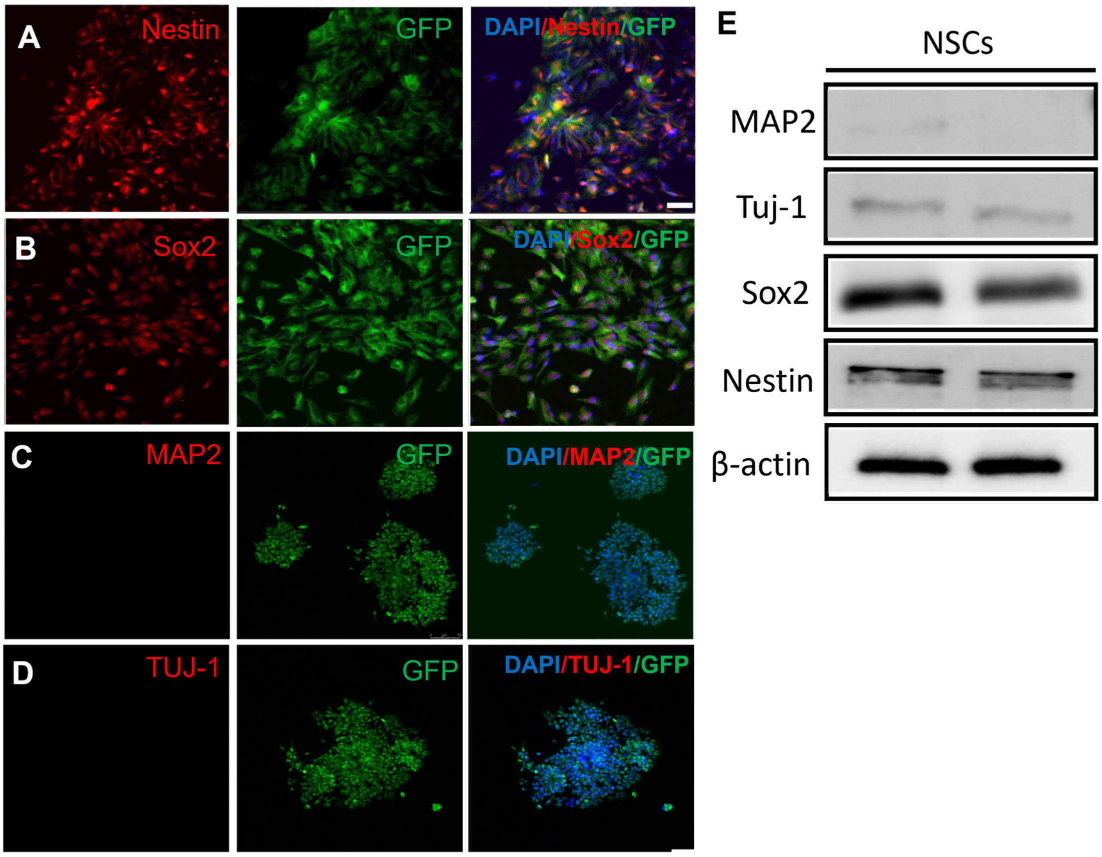

Figure S1. Immunofluorescence images of NSCs before transplantation.

(A) NSCs (GFP) expressed characteristic NSC marker (Nestin, red).

(B) NSCs (GFP) expressed characteristic NSC marker (Sox2, red).

(C-D) NSCs (GFP) were negative for neural markers (MAP2/Tuj-1, red). Scale bars, 50 μm.

(E) According to western blot analysis, NSCs were negative for neural markers (MAP2/Tuj-1) and expressed characteristic NSC markers (SOX2/Nestin).

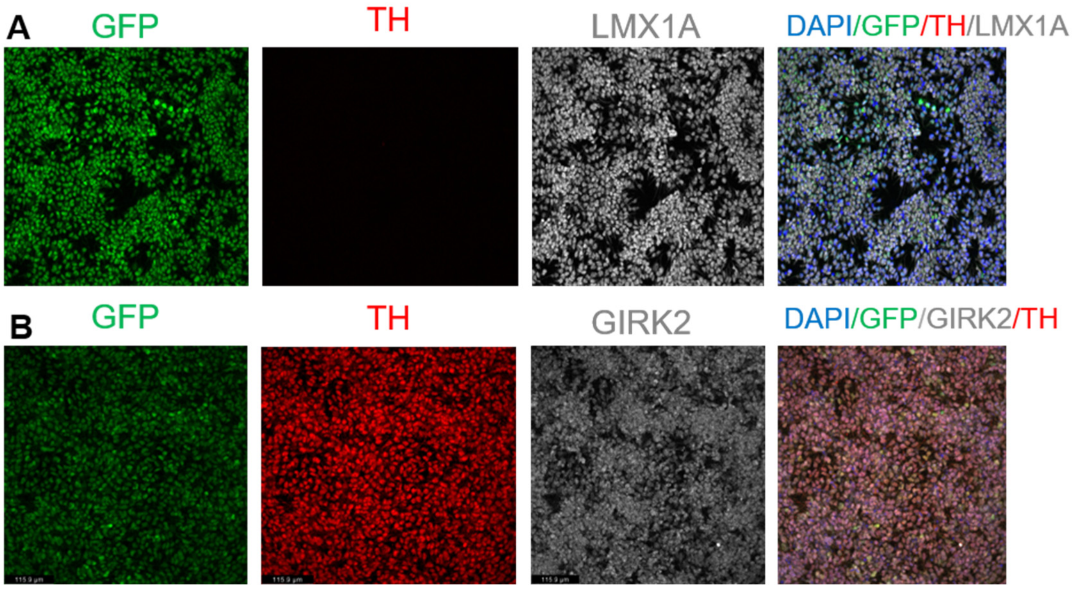

Figure S2. NSCs could differentiate into DA neurons (TH/GIRK2).

(A) NSCs (GFP) expressed a DA progenitor marker (LMX1A, grey).

(B) The differentiated NSCs (GFP) expressed mature DA neuron markers (TH, red/GIRK2, grey). Scale bars, 50 μm.

Immunogen	Species	Cat. No	Supplier	Dilution for ICC
Tuj-1	Mouse	MAB1637	Millipore	1:200
NF	Rabbit	AB207176	Abcam	1:500
NeuN	Mouse	MAB377	Millipore	1:200
c-Fos	Rabbit	226003	Synaptic system	1:200
Synapsin	Rabbit	S193	sigma	1:800
PSD-95	Mouse	Ab2723	Abcam	1:500
GFAP	Mouse	SMI-21R	BioLegend	1:1000
TH	Rabbit	AB137869	Abcam	1:500
Nestin	Rabbit	AB5922	Millipore	1:200
MAP2	Mouse	M4403	Sigma	1:800
LMX1A	Rabbit	NBP2-41193	Novus	1:500
Girk2	Goat	NB100-74575	Novus	1:500

Table S1. Related to Figure 1, 2, 3, 4, 5 and Figure S1, 2. Primary antibodies in alphabetical order.

Name	Supplier	Dilution
Goat anti-Mouse IgG H&L (Alexa Fluor@ 594)	Thermofisher	1:500
Goat anti-Rabbit IgG H&L (Alexa Fluor@ 594)	Thermofisher	1:500
Goat Anti-Rabbit IgG H&L (Alexa Fluor® 647)	Abcam	1:500
Donkey Anti-Goat IgG H&L (Alexa Fluor® 647)	Abcam	1:500

Table S2. Related to Figure 1, 2, 3, 4, 5 and Figure S1, 2. Secondary antibodies.
